# Publisher Correction: Dispersal homogenizes communities via immigration even at low rates in a simplified synthetic bacterial metacommunity

**DOI:** 10.1038/s41467-019-09885-5

**Published:** 2019-04-25

**Authors:** Stilianos Fodelianakis, Alexander Lorz, Adriana Valenzuela-Cuevas, Alan Barozzi, Jenny Marie Booth, Daniele Daffonchio

**Affiliations:** 1 Biological and Environmental Sciences and Engineering Division (BESE), Red Sea Research Center, King Abdullah University of Science and Technology (KAUST), Thuwal, 23955-6900 Saudi Arabia; 2 Computer Electrical and Mathematical Science and Engineering Division (CEMSE), King Abdullah University of Science and Technology (KAUST), Thuwal, 23955-6900 Saudi Arabia

**Keywords:** Microbial ecology, Microbial ecology

Correction to: *Nature Communications* 10.1038/s41467-019-09306-7, published online 21 March 2019

In the original version of this article, the green and blue outlines in Fig. 2b, top centre and right panels were inadvertently shifted left from the correct position. This has now been corrected in the PDF and HTML versions of the article.

