# Supplementary material for: Dispersal homogenizes communities via immigration even at low rates in a simplified synthetic bacterial metacommunity
Source: Nat Commun. 2019 Mar 21;10:1314. doi: 10.1038/s41467-019-09306-7 (PMC6428813; doi:10.1038/s41467-019-09306-7)
Supplement: Supplementary file 1 — Supplementary Information [file 41467_2019_9306_MOESM1_ESM.pdf]

Supplementary Information

Dispersal homogenizes communities via immigration even at low rates in a simplified synthetic bacterial metacommunity

Fodelianakis et al.

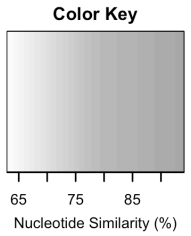

Pairwise Nucleotidic Similarity of Representative Isolates

	A2	A4	A7	A9	A13	A30	A32	A47	B18	B42	C15	D3	D11	D23	E53	E58	E70	E111	E137	E245	E248	E256	E267	E275	E279	E283	E285	E286	E302	E305	E310
A2		85	85	81	81	77	70	83	82	65	72	70	78	73	82	73	80	70	80	72	79	70	69	73	78	70	76	68	72	65	69
A4	85		93	79	80	75	69	83	80	64	72	70	78	72	81	73	80	70	79	71	78	70	70	72	77	70	74	69	71	66	70
A7	85	93		80	82	74	71	83	80	65	72	70	76	72	79	73	80	71	80	73	77	71	69	73	76	69	74	69	72	66	70
A9	81	79	80		82	76	70	77	83	67	72	69	78	74	77	74	82	71	82	74	79	69	69	72	79	69	75	68	74	67	69
A13	81	80	82	82		77	74	78	92	67	75	71	81	75	78	76	93	73	89	74	81	71	71	74	79	71	76	71	74	67	72
A30	77	75	74	76	77		68	74	77	65	70	69	79	72	74	73	76	69	77	71	78	69	69	72	86	69	81	68	72	65	68
A32	70	69	71	70	74	68		69	71	65	87	73	70	71	67	90	72	85	72	71	72	69	71	72	70	71	67	71	72	63	85
A47	83	83	83	77	78	74	69		78	66	70	68	76	71	80	72	78	68	77	71	76	68	68	71	76	68	73	68	71	65	69
B18	82	80	80	83	92	77	71	78		67	75	72	80	76	79	77	93	74	91	75	81	71	71	74	80	72	77	71	74	67	74
B42	65	64	65	67	67	65	65	66	67		67	67	69	66	64	69	67	67	66	66	68	65	67	69	69	66	66	67	67	70	66
C15	72	72	72	72	75	70	87	70	75	67		76	74	73	70	92	75	91	75	73	74	72	74	75	73	73	71	73	73	66	87
D3	70	70	70	69	71	69	73	68	72	67	76		73	72	69	76	71	73	71	71	73	84	90	85	73	88	69	85	71	66	73
D11	78	78	76	78	81	79	70	76	80	69	74	73		76	76	76	81	73	80	74	93	72	72	76	81	72	78	72	74	68	71
D23	73	72	72	74	75	72	71	71	76	66	73	72	76		70	75	75	73	75	90	75	72	71	73	74	72	72	70	91	67	72
E53	82	81	79	77	78	74	67	80	79	64	70	69	76	70		72	80	70	78	70	77	69	68	71	77	69	72	68	71	65	68
E58	73	73	73	74	76	73	90	72	77	69	92	76	76	75	72		76	94	76	75	76	73	75	74	73	74	73	73	76	69	88
E70	80	80	80	82	93	76	72	78	93	67	75	71	81	75	80	76		74	91	74	82	71	70	76	79	72	77	71	73	67	73
E111	70	70	71	71	73	69	85	68	74	67	91	73	73	73	70	94	74		74	72	74	70	73	75	73	72	69	72	73	66	86
E137	80	79	80	82	89	77	72	77	91	66	75	71	80	75	78	76	91	74		74	81	70	70	74	81	70	77	69	74	66	72
E245	72	71	73	74	74	71	71	71	75	66	73	71	74	90	70	75	74	72	74		73	70	70	72	74	72	71	69	88	67	71
E248	79	78	77	79	81	78	72	76	81	68	74	73	93	75	77	76	82	74	81	73		72	72	76	81	73	77	72	74	67	72
E256	70	70	71	69	71	69	69	68	71	65	72	84	72	72	69	73	71	70	70	70	72		86	84	72	84	69	83	71	66	70
E267	69	70	69	69	71	69	71	68	71	67	74	90	72	71	68	75	70	73	70	70	72	86		87	71	87	68	86	71	66	73
E275	73	72	73	72	74	72	72	71	74	69	75	85	76	73	71	74	76	75	74	72	76	84	87		72	87	73	90	72	70	74
E279	78	77	76	79	79	86	70	76	80	69	73	73	81	74	77	73	79	73	81	74	81	72	71	72		73	84	71	75	68	72
E283	70	70	69	69	71	69	71	68	72	66	73	88	72	72	69	74	72	72	70	72	73	84	87	87	73		69	86	72	66	73
E285	76	74	74	75	76	81	67	73	77	66	71	69	78	72	72	73	77	69	77	71	77	69	68	73	84	69		69	72	66	68
E286	68	69	69	68	71	68	71	68	71	67	73	85	72	70	68	73	71	72	69	69	72	83	86	90	71	86	69		70	66	72
E302	72	71	72	74	74	72	72	71	74	67	73	71	74	91	71	76	73	73	74	88	74	71	71	72	75	72	72	70		67	72
E305	65	66	66	67	67	65	63	65	67	70	66	66	68	67	65	69	67	66	66	67	67	66	66	70	68	66	66	66	67		65
E310	69	70	70	69	72	68	85	69	74	66	87	73	71	72	68	88	73	86	72	71	72	70	73	74	72	73	68	72	72	65	

Supplementary Figure 1. Pairwise nucleotidic similarity heatmap based on comparisons of the 16S rRNA gene sequences of the 31 OTUs that were attributed to the 317 isolates of our collection. The code of the representative isolates is given on the right of the rows and on the bottom of the columns. The higher the similarity, the darker the color of each cell as per the legend at the top left. The exact percentages of similarity are also shown within each cell.

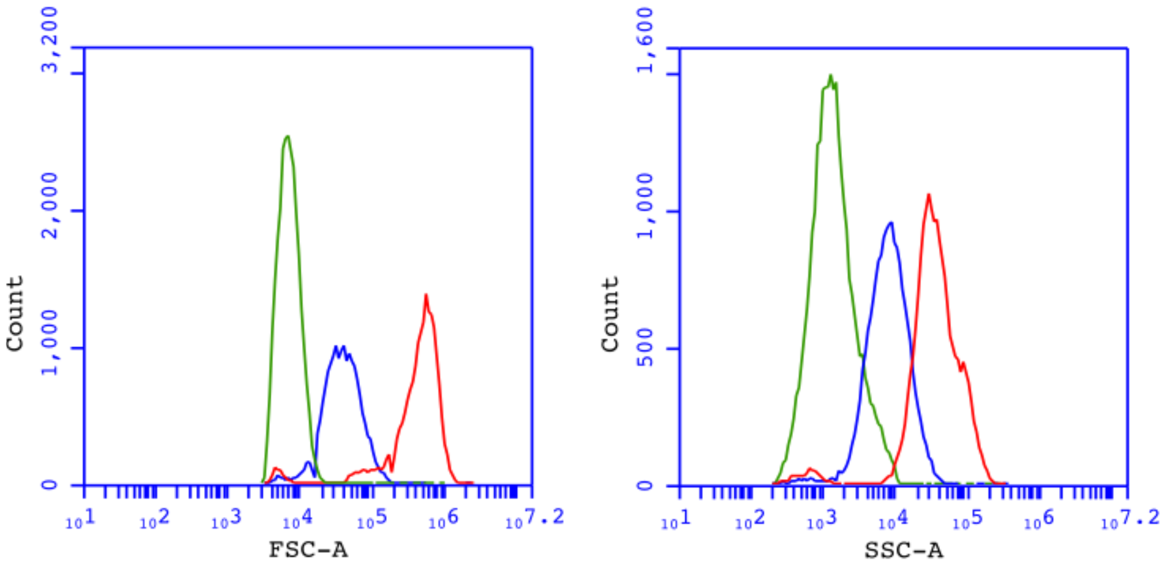

Supplementary Figure 2. Histograms showing the distribution of the forward-scatter area (left plot) and side-scatter area (right plot) signals of the three strains that we selected to assemble our synthetic community. The green line corresponds to strain B42 (*Chryseobacterium* sp.), the blue line corresponds to strain E310 (*Staphylococcus* sp.) and the red line corresponds to strain E111 (*Bacillus* sp.). The y-axis shows the number of recorded events and the x-axis shows the arbitrary units of events recorded with a BD Accuri C6 cytometer with standard settings. The shown values were recorded from 20.5 µl of a 1,000-fold diluted overnight pure culture for each strain at an acquisition speed of 14 µl per min.

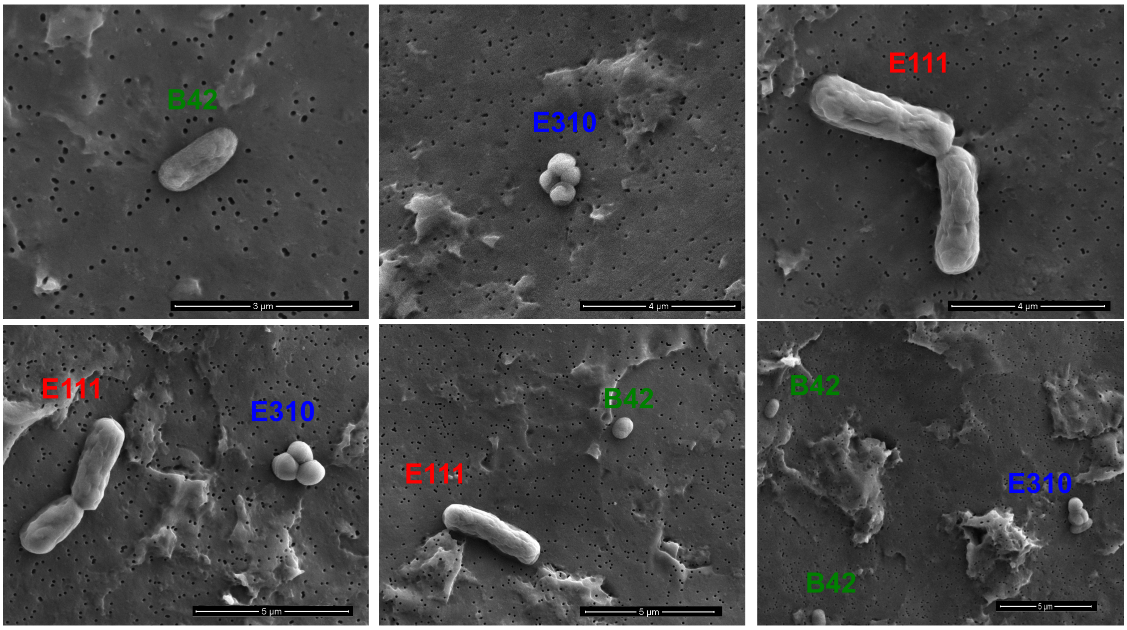

Supplementary Figure 3. Scanning electron microscopy (SEM) images of the three bacteria, fixed on a 0.22 um polycarbonate filter. E111: *Bacillus sp.*, E310: *Staphylococcus sp.,* B42: *Chryseobacterium sp.* The top three images (left to right) are taken from pure cultures of each strain and the three bottom images (left to right) are taken from mixed cultures of E111/E310, E111/B42 and B42/E310, respectively. The scale bars were adjusted from the original SEM image.

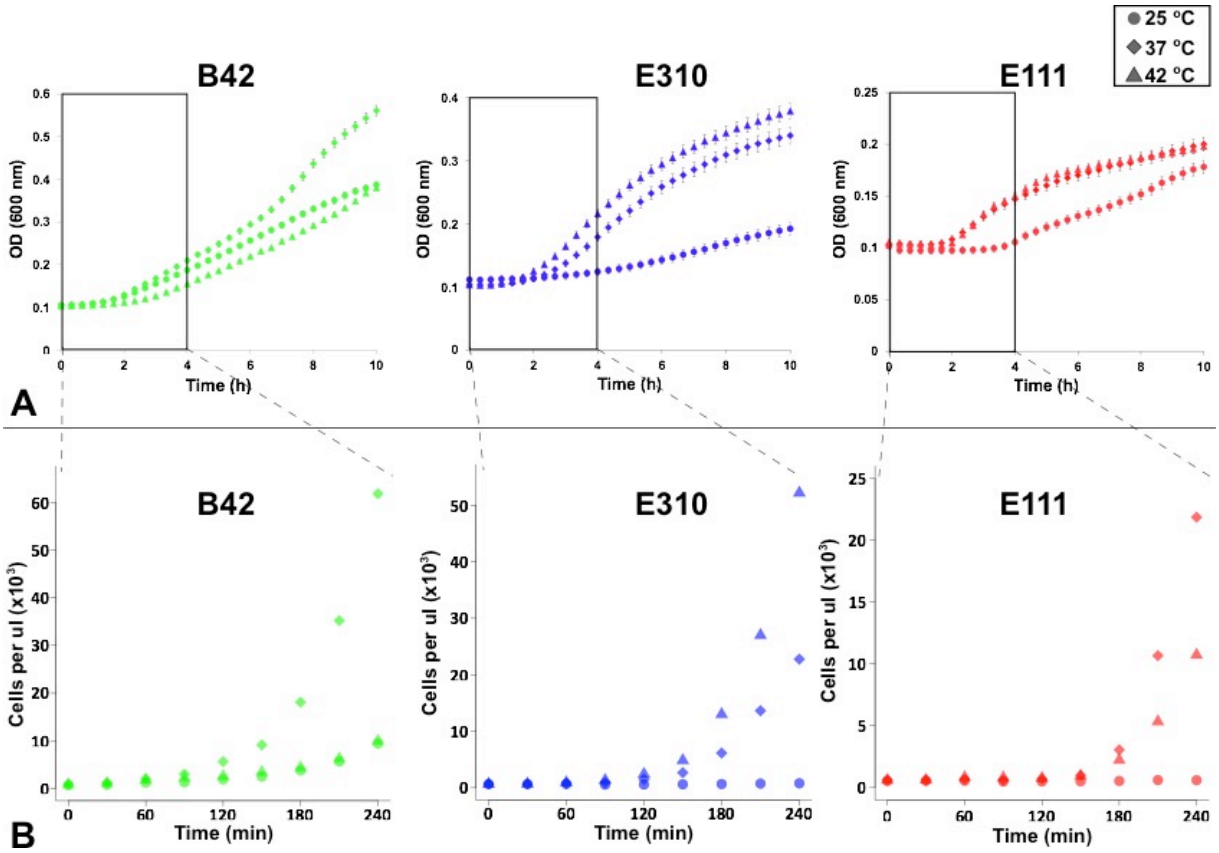

Supplementary Figure 4. The growth of pure cultures of each of the three strains that we selected to comprise the synthetic community, at three different nominal temperatures. The nominal incubation temperatures are represented by different symbols as shown on the legend at the top right. **A.** The growth of each strain in each different temperature during a ten-hour incubation, determined spectrophotometrically by the increase in the optical density of the cultures at 600 nm. Error bars represent one standard error, derived from six biological replicates. Rectangles represent the first four hours of incubation where the population densities of each strain at each temperature were further measured using the method based on staining-free flow cytometry that we developed. **B.** The growth of the pure cultures at each temperature during a four-hour incubation determined by staining-free flow cytometry. The starting cell densities were 666-836 cells μl^{-1}, 633-699 cells μl^{-1} and 548-616 cells μl^{-1} for strains B42, E310 and E111, respectively, to be comparable to the total starting cell densities at the main experiment. The experimental setup and the sampling strategy was the same that we used for the mixed cultures (both in the experiments with and in the experiments without dispersal) as described in the main text. The mean values of three biological replicates are shown, and standard deviations were low (<3.5% of the mean values) and were excluded to assist visualization.

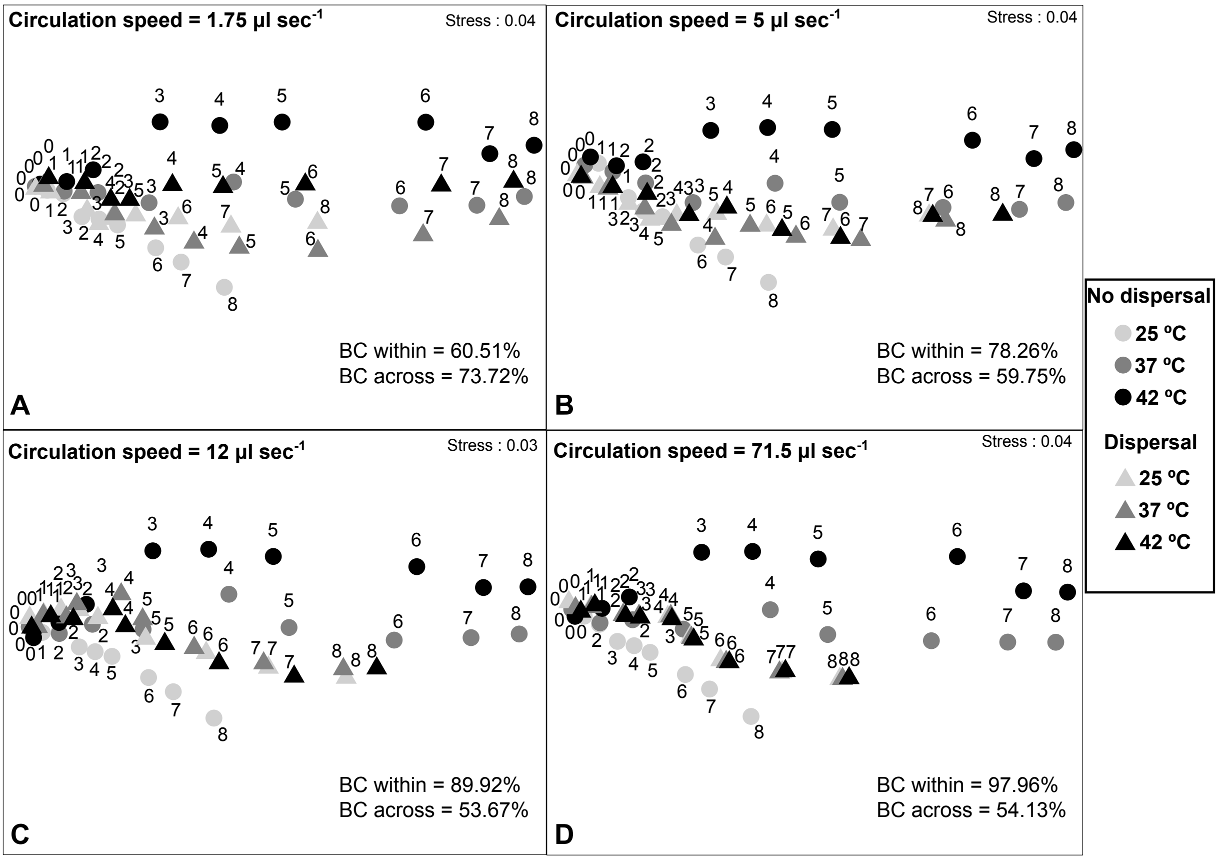

Supplementary Figure 5. Non-metric multidimensional plots of the synthetic communities in the experiments with different circulation speed. Each panel contains communities from an experiment at a given circulation speed (triangles) and communities from the experiment without dispersal (circles) for comparison. Panels A, B, C and D contain communities without dispersal and communities from experiments at circulation speeds of 1.75, 5, 12 and 71.5 µl sec^{-1}, respectively. Each symbol stands for the centroid from three biological replicates (no dispersal) or three independent experiments (dispersal). Numbers above the samples represent the sampling time with 30 min intervals, with 0 being the beginning of the experiment and 8 the end. "BC within" represents the average Bray-Curtis similarity (BC) within the metacommunity (triangle-triangle, same time). "BC across" represents the average BC among communities growing under a given degree of dispersal compared to the respective communities growing at the same temperature but without dispersal (circle-triangle, same time and color).

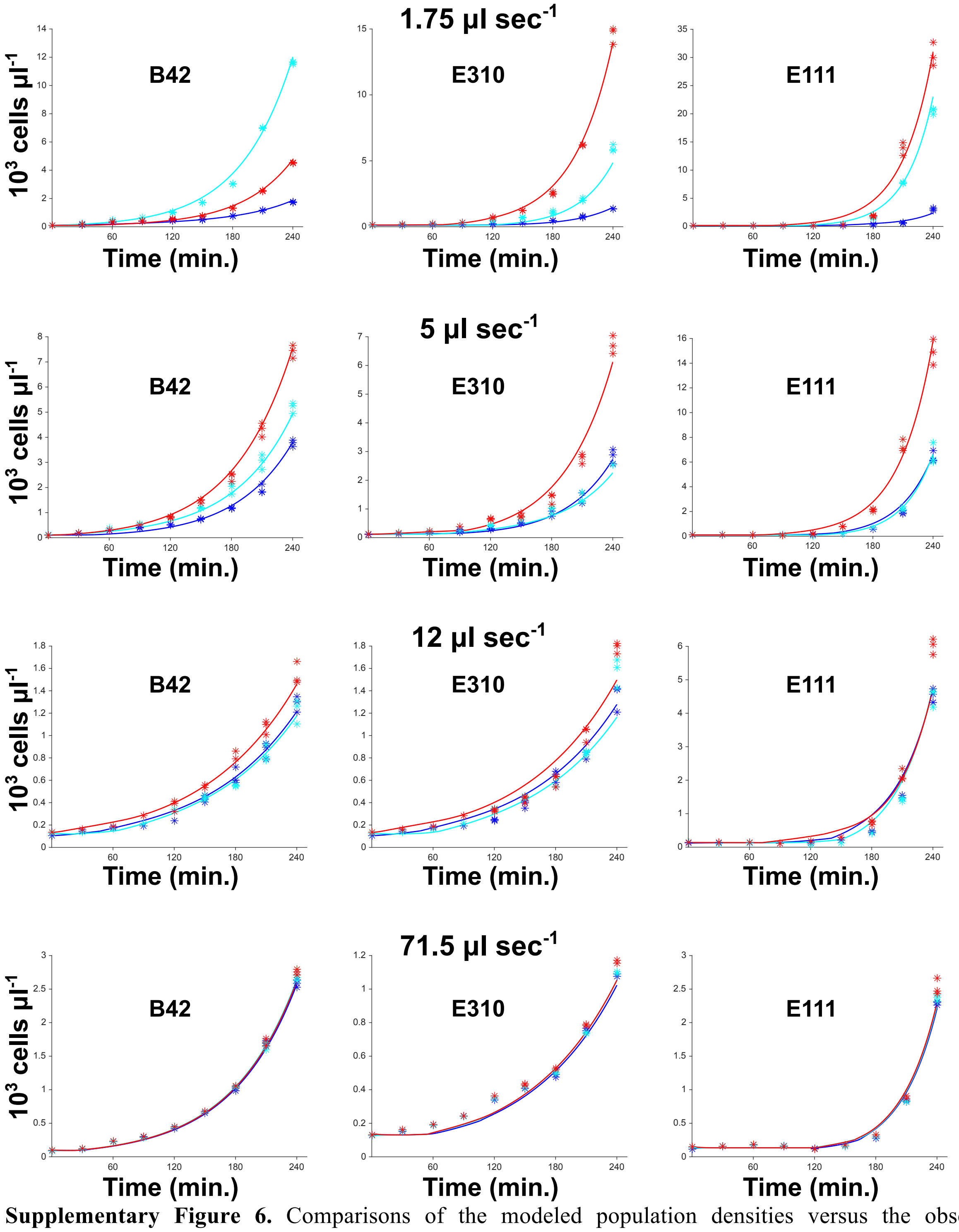

Supplementary Figure 6. Comparisons of the modeled population densities versus the observed population densities in the experiments with dispersal. The population density is shown on the y-axis and the experimental time is shown on the x-axis. The lines in each plot correspond to the modeled population densities and the asterisks correspond to the observed population densities from three independent experiments. Blue stands for communities at nominal incubation temperature of 25°C, cyan for communities at 37°C and red for communities at 42°C.

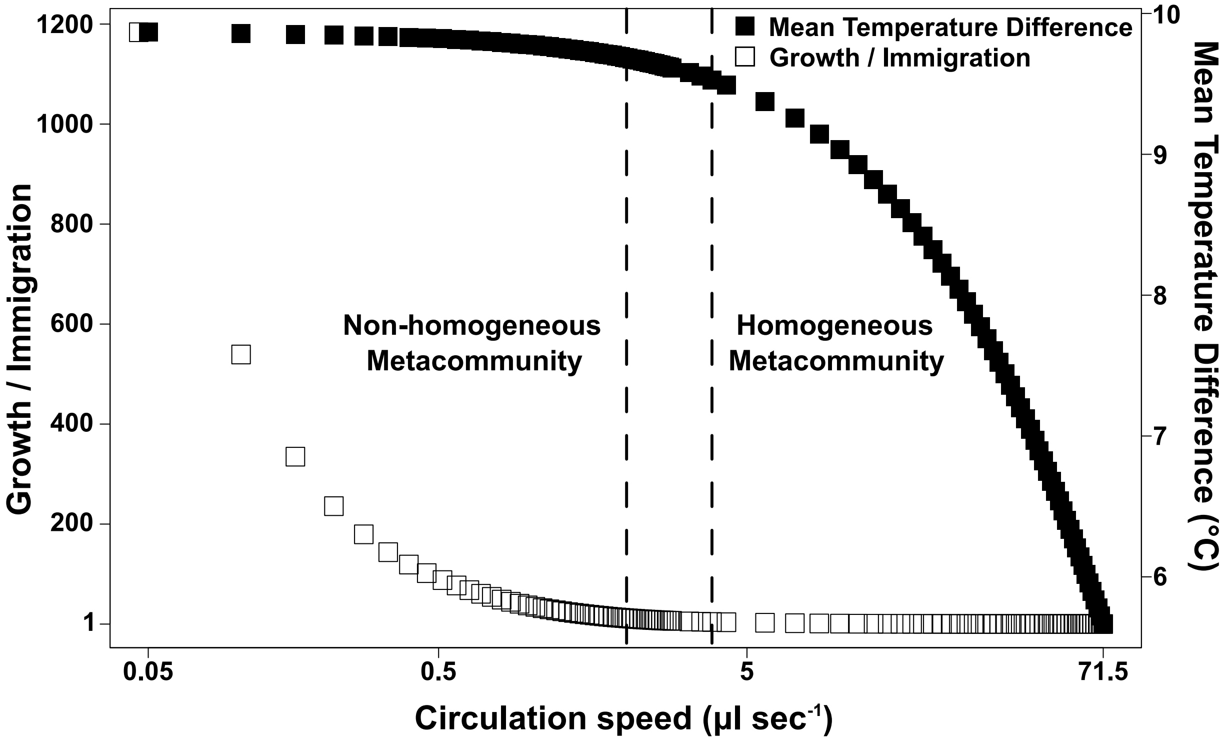

Supplementary Figure 7. The growth-over-immigration ratio (left side) and the mean temperature difference among the three incubation vessels (right side) as a function of increasing circulation speed (in logarithmic scale). The vertical dashed lines are drawn based on Figure 5A.

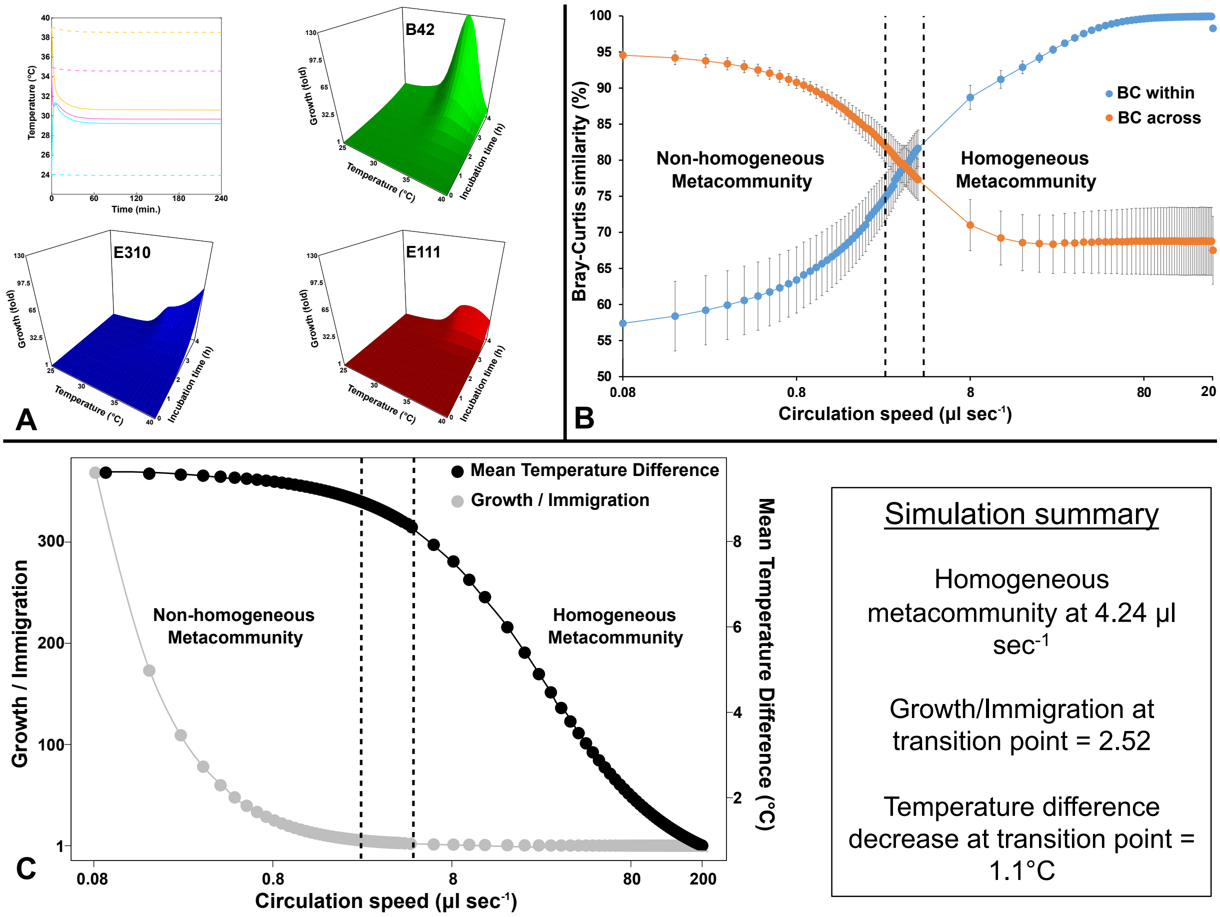

Supplementary Figure 8. A summary of the properties and the results of the first additional simulation. **A.** The modeled changes in temperature over time (upper left) and in the growth of each strain (upper right and below) in the simulations with increasing circulation speed. Regarding temperature, the dashed curves represent the lowest circulation speed (0.08 µl sec^{-1}) and the solid curves the highest circulation speed (200 µl sec^{-1}). Cyan represents vessels at nominal incubation temperature of 25°C, yellow vessels at 37°C and magenta vessels at 42°C. Regarding growth, temperature is given on the x-axis, time is given on the y-axis and growth is given on the z-axis. **B.** Bray-Curtis similarity as a function of increasing circulation speed (in logarithmic scale). Vertical dashed lines delimit the range of circulation speed under which "BC within" is significantly lower than "BC across" and vice versa (Mann-Whitney tests, two-sided $p < 0.05$). Vertical bars represent one standard error (n=21). **C.** The growth-over-immigration ratio (left side) and the mean temperature difference among the three vessels (right side) as a function of increasing circulation speed. The vertical dashed lines are drawn based on panel B.

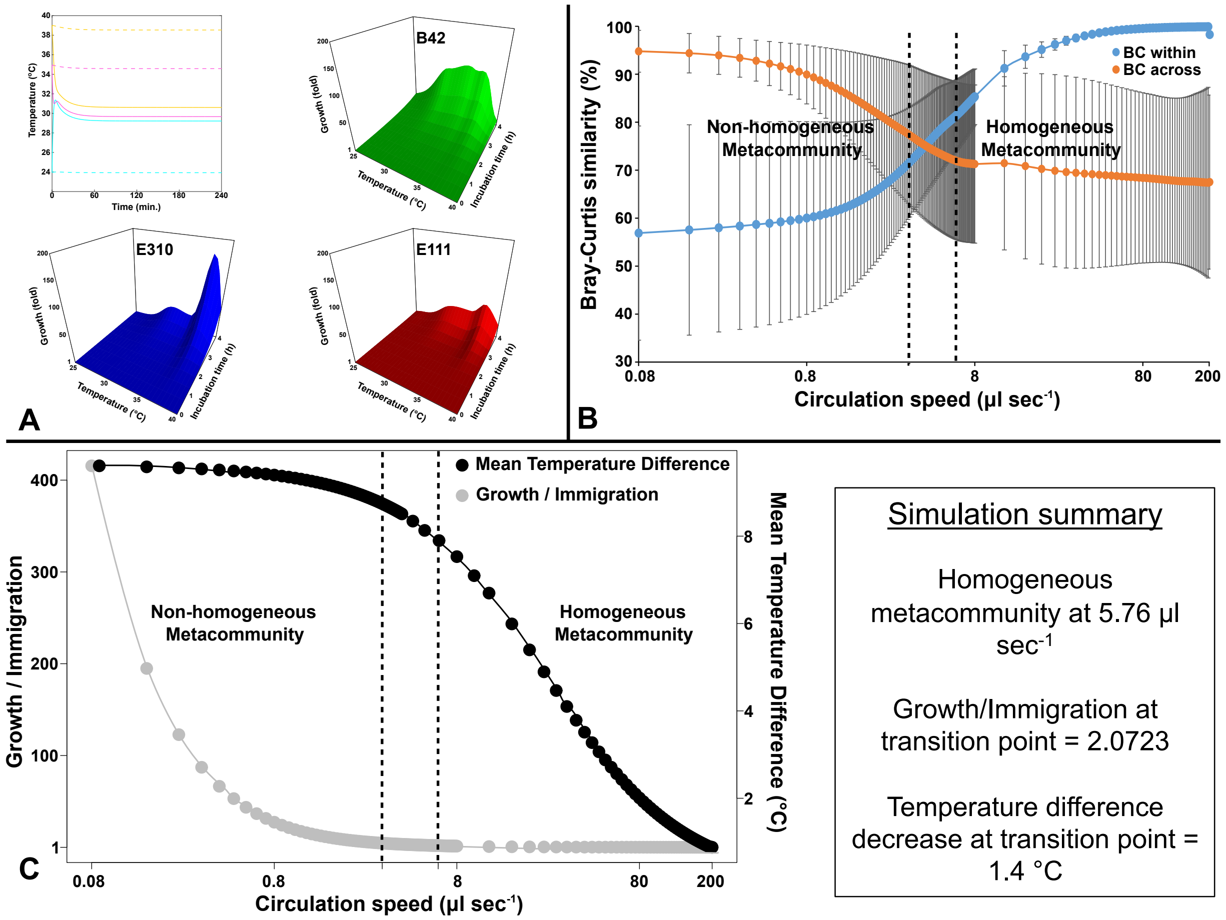

Supplementary Figure 9. A summary of the properties and the results of the second additional simulation. **A.** The modeled changes in temperature over time (upper left) and in the growth of each strain (upper right and below) in the simulations with increasing circulation speed. Regarding temperature, the dashed curves represent the lowest circulation speed (0.08 µl sec^{-1}) and the solid curves the highest circulation speed (200 µl sec^{-1}). Cyan represents vessels at nominal incubation temperature of 25°C, yellow vessels at 37°C and magenta vessels at 42°C. Regarding growth, temperature is given on the x-axis, time is given on the y-axis and growth is given on the z-axis. **B**. Bray-Curtis similarity as a function of increasing circulation speed (in logarithmic scale). Vertical dashed lines delimit the range of circulation speed under which "BC within" is significantly lower than "BC across" and vice versa (Mann-Whitney tests, two-sided $p < 0.05$). Vertical bars represent one standard deviation (n=21). **C.** The growth-over-immigration ratio (left side) and the mean temperature difference among the three vessels (right side) as a function of increasing circulation speed. The vertical dashed lines are drawn based on panel B.

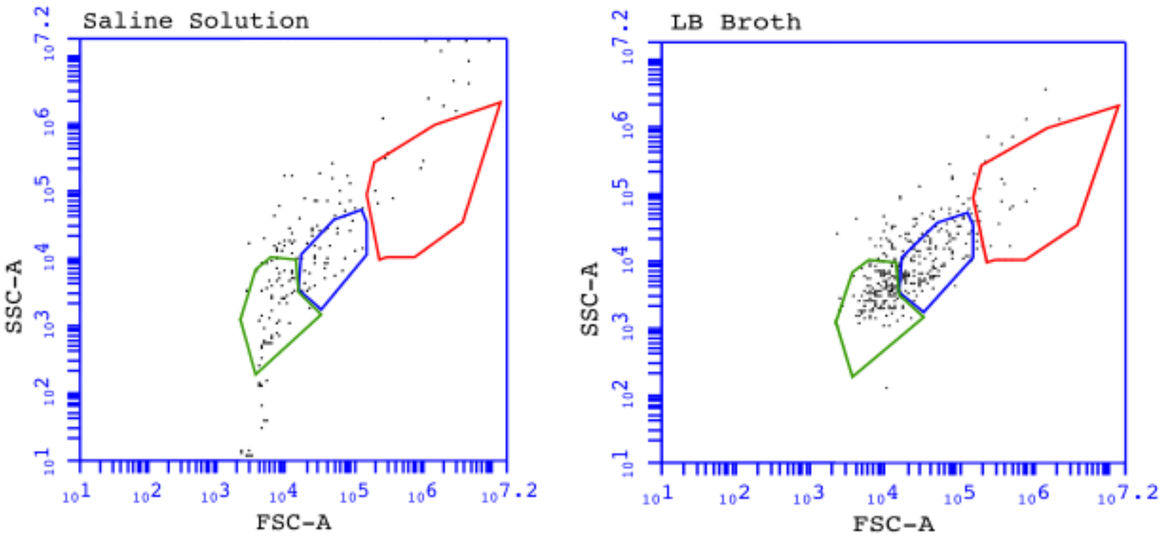

Supplementary Figure 10. The scattering profiles of the particles within the sterile physiological solution that we used to dilute cultures when needed (left side) and within the sterile LB broth where we incubated our synthetic community (right side). The "representative" gates for each of the three strains of the synthetic community at the beginning of the incubation are also drawn for visual assistance (green for B42, blue for E310 and red for E111). The shown profiles were recorded from 20.5 µl of each solution at an acquisition speed of 14 µl per min. For the sterile physiological solution the average particle count was 4.54 events per µl in total. For the sterile LB broth, the average particle count per µl at each gate was 15.2 at the gate of B42, 4.9 at the gate of E310 and 1.01 at the gate of E111. Those counts were deducted from the observed counts prior to the analyses.

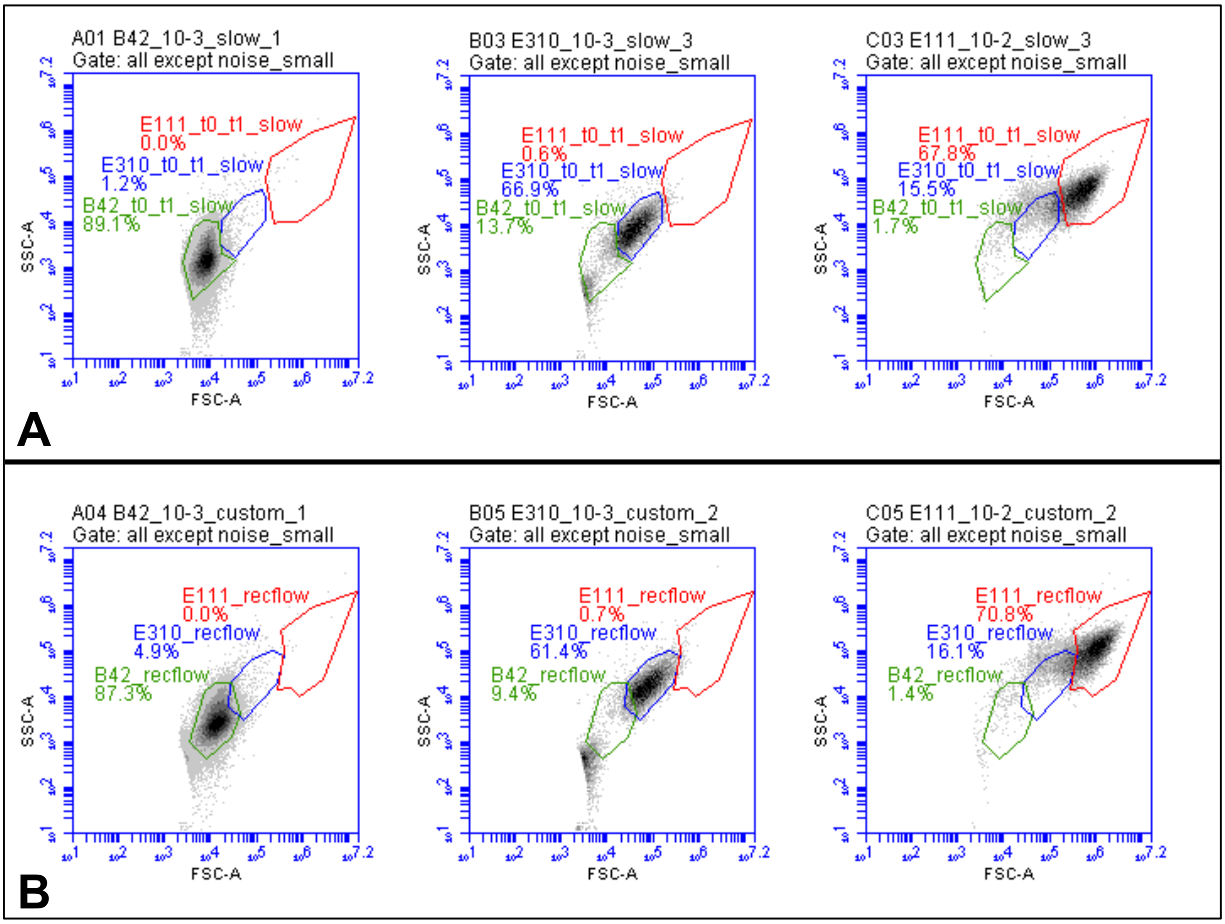

Supplementary Figure 11. Typical FSC-A / SSC-A profiles of overnight cultures of the three pure strain using the "Slow" (A) and "minimum recommended" (B) settings of the BD Accuri C6. The shown profiles were acquired by sampling the same volume, 20 µl.

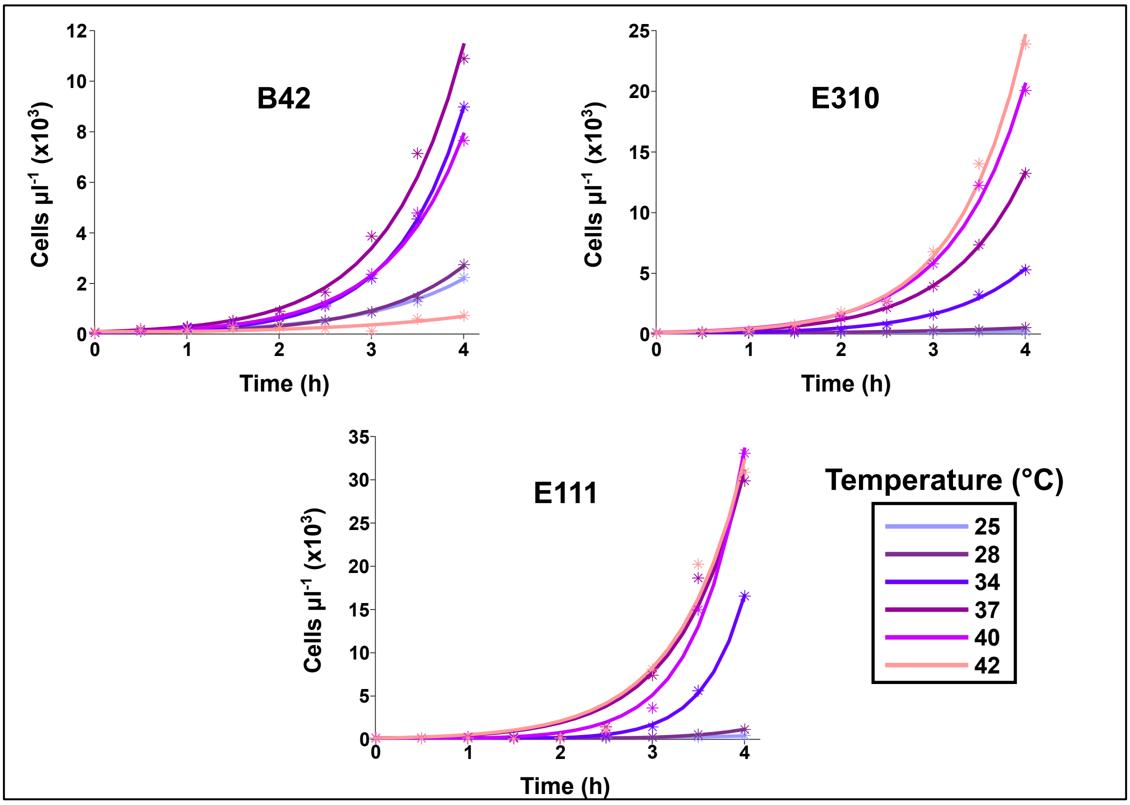

Supplementary Figure 12. Comparisons of the modeled population densities versus the observed population densities in the experiments without dispersal. The population density is shown on the y-axis and the experimental time is shown on the x-axis. The lines in each plot correspond to the modeled population densities and the asterisks correspond to the average observed population densities from three biological replicates. The color of the lines and the asterisks corresponds to the legend on the bottom right, where the nominal temperature of incubation is given next to each line.

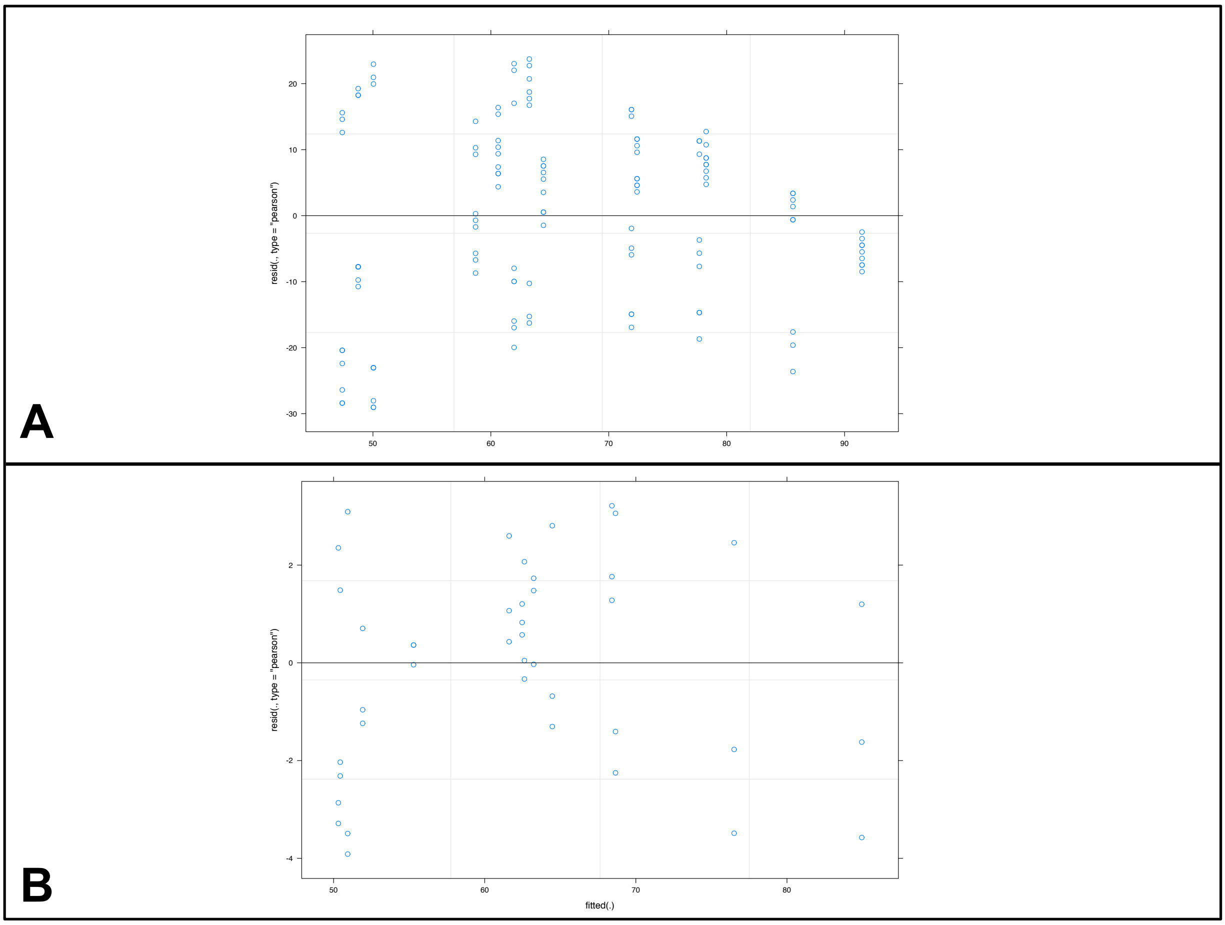

Supplementary Figure 13. Plots of the fitted residuals from a typical linear mixed effects model (A – experimental data from the experiment with dispersal of 1.75 µl sec^{-1}) and from a typical generalized linear mixed effects model (B – modeled data from the scenario with dispersal of 1.83 µl sec^{-1}) for the comparison of “BC within” and “BC across”.

Supplementary Table 1. The taxonomic affiliation of the 31 OTUs that were formed from the 317 isolates of our collection. The first column shows the assigned OTU number, the second column shows the code of the representative isolate, the third column shows the taxonomic affiliation of best BLAST hit from the "refseq_rRNA" database of NCBI, the fourth column shows the percentage of nucleotide similarity between the best BLAST hit and the representative sequence of each OTU and the fifth column shows the number of isolates that clustered in each OTU. Rows in bold represent the three representative isolates that we chose to assemble the synthetic community.

OTU	Representative isolate code	Closest Cultured Representative *(reference sequence)*	Nucleotide Identity (%)	# of isolates
1	A2	*Zobellella taiwanensis strain ZT1* (NR_043630)*	97%	12
2	A4	*Enterobacter cloacae strain DSM 30054* (NR_117679.1)*	99%	1
3	A7	*Siccibacter colletis strain 1383* (NR_134807)	99%	2
4	A9	*Acinetobacter venetianus strain ATCC 31012* (NR_042049)	99%	1
5	A13	*Pseudomonas plecoglossicida strain NBRC 103162* (NR_114226)*	99%	24
6	A30	*Cupriavidus plantarum strain ASC-64* (NR_109160)*	99%	1
7	A32	*Lysinibacillus pakistanensis strain NCCP-54* (NR_113166)*	99%	1
8	A47	*Photobacterium galatheae strain S2753* (NR_145878)	99%	1
9	B18	*Pseudomonas mendocina strain ATCC 25411* (NR_114477)*	99%	26
**10**	**B42**	***Chryseobacterium indologenes strain NBRC 14944* (NR_112975)**	**99%**	**1**
11	C15	*Bacillus cereus strain ATCC 14579* (NR_074540)	99%	46
12	D3	*Arthrobacter endophyticus strain EGI 6500322* (NR_136480)	99%	11
13	D11	*Pseudoxanthomonas japonensis strain NBRC 101033* (NR_113972)*	99%	1
14	D23	*Ensifer adhaerens strain NBRC 100388* (NR_113893)*	99%	1
15	E53	*Aeromonas taiwanensis strain A2-50* (NR_116585)*	99%	5
16	E58	*Bacillus koreensis strain BR030* (NR_043084)	99%	45
17	E70	*Pseudomonas mosselii strain CFML 90-83* (NR_024924)*	99%	21
**18**	**E111**	***Bacillus megaterium strain NBRC 15308* (NR_112636)***	**99%**	**40**
19	E137	*Pseudomonas resinovorans strain ATCC 14235* (NR_112062)	98%	31
20	E245	*Ochrobactrum haematophilum strain CCUG 38531* (NR_042588)	99%	1
21	E248	*Stenotrophomonas rhizophila strain e-p10* (NR_121739)	99%	3
22	E256	*Rhodococcus equi strain ATCC 6939* (NR_116691)*	99%	8
23	E267	*Arthrobacter enclensis strain NIO-1008* (NR_134699)	99%	12
24	E275	*Microbacterium esteraromaticum strain DSM 8609* (NR_026468)*	99%	4
25	E279	*Achromobacter mucicolens strain R-46658* (NR_117613)*	99%	1
26	E283	*Cellulosimicrobium cellulans strain DSM 43879* (NR_119095)	99%	1
27	E285	*Xenophilus arseniciresistens strain YW8* (NR_133894)	99%	1
28	E286	*Agromyces mediolanus strain VKM Ac-1388* (NR_118453)*	99%	1
29	E302	*Rhizobium pusense strain NRCPB10* (NR_116874)*	99%	10
30	E305	*Sphingobacterium multivorum strain IAM 14316* (NR_040953)*	98%	3
**31**	**E310**	***Staphylococcus epidermidis strain Fussel* (NR_036904)***	**99%**	**1**
			**Total**	**317**

* Multiple hits with the same % of identity to our query sequence were found, and these hits affiliated to different species within the same Genus.

Supplementary Table 2. The forward and side-scattering properties of pure cultures of the 31 representative isolates. The first column shows the assigned code for each representative isolate, the second column shows the mean area value of the forward scatter (FSC-A), the third column shows the coefficient of variation (CV) for the mean FSC-A, the fourth column shows the mean area value of the side scatter (SSC-A) and the fifth column shows the CV for the mean SSC-A. FSC-A and SSC-A values correspond to arbitrary units of events recorded with a BD Accuri C6 flow cytometer with standard settings. The shown values were based on 100,000 events of a pure culture for each strain, recorded at an acquisition speed of 14 µl per min. Rows in bold represent the three representative isolates that we chose to assemble the synthetic community. All samples were acquired from overnight cultures in LB medium.

Strain code	Mean FSC-A	CV FSC-A	Mean SSC-A	CV SSC-A
A2	55,161.08	47.88%	6,393.63	103.19%
A4	11,733	42.79%	2,402.77	83.65%
A7	23,383.63	57.87%	5,209.48	153.87%
A9	11,867.01	45.86%	2,493.66	94.49%
A13	23,077.96	48.06%	3,415.01	112.11%
A30	24,112.51	56.35%	4,954.82	146.31%
A32	74,053.75	61.05%	16,977.83	188.95%
A47	26,930.16	113.55%	3,315.78	91.97%
B18	9,636.55	67.46%	3,922.65	148.99%
**B42**	**6,792.48**	**45.91%**	**2,426.82**	**130.56%**
C15	141,121.02	112.33%	26,732.17	118.13%
D3	17,298.40	63.78%	5,600.19	112.71%
D11	10,319.71	74.74%	5,372.36	145.29%
D23	10,346.77	57.28%	2,762.39	100.13%
E53	10,161.04	61.66%	3,741.71	120.82%
E58	182,956.85	60.13%	17,121.93	137.57%
E70	13,321.09	48.20%	3,791.67	116.53%
**E111**	**808,873.76**	**43.53%**	**76,974.97**	**65.59%**
E137	18,423.85	51.09%	4,995.56	112.39%
E245	8,808.72	54.64%	2,051.51	98.48%
E248	9,842.42	64.90%	3,847.93	123.18%
E256	38,244.29	85.20%	10,575.02	100.67%
E267	21,674.12	63.81%	6,422.22	92.89%
E275	11,690.39	93.89%	4,805.07	121.27%
E279	8,469.75	64.49%	3,266.62	142.17%
E283	9,278.55	59.79%	3,961.04	155.58%
E285	12,498.37	63.54%	2,954.22	108.21%
E286	9,418.60	64.29%	3,636.95	110.47%
E302	8,074.06	75.88%	2,973.73	168.06%
E305	15,754.57	67.29%	5,065.87	166.59%
**E310**	**40,203.48**	**71.74%**	**13,905.57**	**75.19%**

Supplementary Table 3. The percentage of total events (excluding noise) from samples of pure cultures of each isolate that fell within the corresponding "representative" gates at each of the three tested temperatures (25, 37 and 42°C) during a four-hour incubation and sampled every 30 min.

	25 °C			37 °C			42 °C		
**Time (min.)**	**B42**	**E310**	**E111**	**B42**	**E310**	**E111**	**B42**	**E310**	**E111**
**0**	76.3%	73.6%	68.9%	76.2%	71.7%	64.5%	82.3%	73.9%	65.0%
**30**	78.5%	76.2%	70.6%	78.0%	79.0%	67.8%	80.3%	77.8%	60.9%
**60**	71.8%	76.3%	69.5%	74.2%	79.0%	61.4%	71.5%	78.1%	51.4%
**90**	78.9%	84.0%	75.9%	80.9%	77.6%	65.1%	66.1%	70.7%	52.4%
**120**	80.4%	83.8%	73.4%	83.0%	69.0%	65.9%	63.0%	71.6%	54.9%
**150**	78.7%	83.1%	73.5%	82.9%	77.3%	76.3%	56.3%	89.0%	63.5%
**180**	74.5%	71.9%	72.0%	82.0%	91.4%	86.7%	54.6%	93.0%	84.1%
**210**	71.5%	69.3%	70.6%	82.7%	93.0%	81.5%	54.7%	94.5%	80.0%
**240**	59.5%	46.9%	70.4%	83.4%	94.9%	79.8%	51.3%	94.1%	59.9%

Supplementary Table 4. The spillover ratios for growth at 25°C, defined as the ratio of the events within a "representative gate" for the pure culture of a given strain to the events within the "representative gates" of the other two strains. The spillover ratios of B42 to the gate of E111 were always less than 0.0025 so they were set to 0 for simplicity of calculations.

Time (min.)	B42 to E310	B42 to E111	E310 to B42	E310 to E111	E111 to B42	E111 to E310
**0**	0.020	0	0.147	0.007	0.046	0.128
**30**	0.022	0	0.115	0.013	0.061	0.110
**60**	0.025	0	0.106	0.012	0.061	0.128
**90**	0.024	0	0.080	0.017	0.069	0.144
**120**	0.030	0	0.073	0.017	0.069	0.144
**150**	0.037	0	0.073	0.023	0.075	0.148
**180**	0.062	0	0.070	0.031	0.079	0.149
**210**	0.088	0	0.066	0.042	0.079	0.143
**240**	0.121	0	0.062	0.060	0.081	0.142

Supplementary Table 5. The spillover ratios for growth at 37°C, defined as the ratio of the events within a "representative gate" for the pure culture of a given strain to the events within the "representative gates" of the other two strains. The spillover ratios of B42 to the gate of E111 were always less than 0.0025 so they were set to 0 for simplicity of calculations.

Time (min.)	B42 to E310	B42 to E111	E310 to B42	E310 to E111	E111 to B42	E111 to E310
**0**	0.017	0	0.141	0.007	0.065	0.127
**30**	0.023	0	0.089	0.013	0.075	0.121
**60**	0.026	0	0.066	0.038	0.093	0.220
**90**	0.017	0	0.061	0.112	0.095	0.252
**120**	0.019	0	0.054	0.127	0.085	0.237
**150**	0.023	0	0.034	0.177	0.046	0.127
**180**	0.022	0	0.015	0.043	0.013	0.045
**210**	0.022	0	0.014	0.011	0.004	0.159
**240**	0.022	0	0.008	0.005	0.005	0.223

Supplementary Table 6. The spillover ratios for growth at 42°C, defined as the ratio of the events within a "representative gate" for the pure culture of a given strain to the events within the "representative gates" of the other two strains. The spillover ratios of B42 to the gate of E111 were always less than 0.0025 so they were set to 0 for simplicity of calculations.

Time (min.)	B42 to E310	B42 to E111	E310 to B42	E310 to E111	E111 to B42	E111 to E310
**0**	0.017	0	0.141	0.008	0.066	0.128
**30**	0.027	0	0.086	0.021	0.080	0.217
**60**	0.048	0	0.061	0.068	0.123	0.387
**90**	0.091	0	0.052	0.212	0.124	0.412
**120**	0.167	0	0.042	0.113	0.104	0.432
**150**	0.272	0	0.020	0.061	0.063	0.272
**180**	0.310	0	0.016	0.011	0.021	0.068
**210**	0.320	0	0.016	0.003	0.010	0.130
**240**	0.316	0	0.009	0.001	0.005	0.229

Supplementary Table 7. The accuracy of our method to calculate the individual populations of the three strains in mixed cultures based on the scattering profile of each strain in pure culture and on the spillover ratios among the strains. We performed four tests where we measured the population densities of the three strains in pure cultures and then mixed equal volumes of each pure culture and calculated the population densities in the mixed culture. The expected population densities (column 3) refer to counts from the pure cultures and the observed population densities (column 2) refer to the counts from the mixed cultures (after tripling the latter to account for the 3-fold dilution due to mixing). Accuracy is defined as 1 – (|observed – expected|/expected) and is expressed in percentage.

Strain and test	Observed	Expected	Accuracy (%)
**B42 test1**	1369.932	1407.317	97.3
**E310 test1**	234.9	255.463	92.0
**E111 test1**	155.532	160.878	96.7
**B42 test2**	138	148.19	93.1
**E310 test2**	198.78	198	99.6
**E111 test2**	130.05	128	98.4
**B42 test3**	922.8	936.24	98.6
**E310 test3**	413.4	433.8	95.3
**E111 test3**	504.3	533.58	94.5
**B42 test4**	4189	4375.5	95.7
**E310 test4**	903.8	878.01	97.1
**E111 test4**	1391	1498.62	92.8

Supplementary Table 8. The fit (R^2) of the modeled population densities for each of the strains (columns 3-5) compared to the average observed population densities within each incubation vessel at the experiments with dispersal. For all cases n=27, df=2, two-tailed $p < 0.001$.

Speed (µl sec^{-1})	Temp.	B42	E310	E111
**1.75**	**25°C**	0.99244	0.98953	0.95011
	**37°C**	0.99743	0.97546	0.99245
	**42°C**	0.99612	0.99709	0.98412
**5**	**25°C**	0.99530	0.98221	0.98441
	**37°C**	0.9985	0.97547	0.99107
	**42°C**	0.98956	0.97033	0.99023
**12**	**25°C**	0.98219	0.97032	0.97803
	**37°C**	0.97401	0.96901	0.96362
	**42°C**	0.9667	0.93457	0.96113
**71.5**	**25°C**	0.99880	0.98231	0.99926
	**37°C**	0.99960	0.98633	0.99852
	**42°C**	0.99835	0.98111	0.98736

Supplementary Table 9. The recorded and the modeled temperatures (in °C) at each vessel after four hours of incubation. 25C, 37C and 42C stand for incubation vessels at 25°C, 37°C and 42°C, respectively.

**Circulation Speed ($\mu l\ sec^{-1}$)**	**25C**		**37C**		**42C**	
	**Recorded**	**Modeled**	**Recorded**	**Modeled**	**Recorded**	**Modeled**
**1.75**	25.3	25.196	35.5	35.772	40	39.689
**5**	25.5	25.189	35	35.372	38.7	39.144
**12**	25.2	25.175	34	34.605	38	38.098
**71.5**	26	26.000	31	31.000	33	33.660

Supplementary Table 10. The fit (R^2) of the modeled population densities for each of the strains (columns 2-4) compared to the average observed population densities at the six different temperatures without dispersal. For all cases, except where there was no growth, n=9, df=2, two-tailed $p < 0.001$.

Temp.	B42	E310	E111
**25°C**	0.99263	n/a*	n/a*
**28°C**	0.99989	0.99964	0.98466
**34°C**	0.964	0.99945	0.99918
**37°C**	0.98909	0.9994	0.97961
**40°C**	0.99921	0.99346	0.98303
**42°C**	n/a*	0.99468	0.97216

*non-applicable because the average population density was constant (no growth)
