# Supplementary material for: Dispersal homogenizes communities via immigration even at low rates in a simplified synthetic bacterial metacommunity
Source: Nat Commun. 2019 Mar 21;10:1314. doi: 10.1038/s41467-019-09306-7 (PMC6428813; doi:10.1038/s41467-019-09306-7)
Supplement: Supplementary file 3 — Description of Additional Supplementary Files [file 41467_2019_9306_MOESM3_ESM.pdf]

Description of Additional Supplementary Files

File Name: Supplementary Data 1
Description: An Excel spreadsheet with the comparisons between the observed cell densities of each strain in the incubation vessels with nominal starting temperatures of 25°C, 37°C and 42°C, in pure cultures (column B) and in mixed cultures (column C). Columns H-K report the ANCOVA statistics regarding the comparison of the growth rates between pure and mixed cultures; column H reports the F-ratio, column I the p-value, column J the fitted slope in monocultures and column K the fitted slope in mixed cultures. The file contains six tabs, one for each temperature for experiments starting from similar 'per-strain' (tabs 1-3) and total (tabs 4-6) cell densities.

File Name: Supplementary Data 2
Description: An Excel spreadsheet with the modeled growth rate (r) of each strain in the incubation vessels with nominal starting temperatures of 25°C, 37°C and 42°C (without penalty subtracted), at the 100 scenarios with intermediate circulation speeds. Column A reports the circulation speed, columns B-D, G-I and L-N report the r for strain B42, E310 and E111, respectively. Columns E, J, O report the standard deviation in r among the three vessels and columns F, K and P report the coefficient of variation (%) for strain B42, E310 and E111, respectively.

File Name: Supplementary Movie 1
Description: A movie of forward scatter area (FSC-A – x axis) / side scatter area (SSC-A – y axis) plots (in log-log scale) from strain B42 during a time period of four hours, taken every 30 min. The colored lines delineate the "representative" gates of each strain; green for B42, blue for E310 and red for E111.

File Name: Supplementary Movie 2
Description: A movie of forward scatter area (FSC-A – x axis) / side scatter area (SSC-A – y axis) plots (in log-log scale) from strain E310 during a time period of four hours, taken every 30 min. The colored lines delineate the "representative" gates of each strain; green for B42, blue for E310 and red for E111.

File Name: Supplementary Movie 3
Description: A movie of forward scatter area (FSC-A – x axis) / side scatter area (SSC-A – y axis) plots (in loglog scale) from strain E111 during a time period of four hours, taken every 30 min. The colored lines delineate the "representative" gates of each strain; green for B42, blue for E310 and red for E111.
