# Supplementary material for: Dispersal homogenizes communities via immigration even at low rates in a simplified synthetic bacterial metacommunity
Source: Nat Commun. 2019 Mar 21;10:1314. doi: 10.1038/s41467-019-09306-7 (PMC6428813; doi:10.1038/s41467-019-09306-7)
Supplement: Supplementary file 9 — Reporting Summary [file 41467_2019_9306_MOESM9_ESM.pdf]

nature research

Corresponding author(s): Daniele Daffonchio, Stilianos Fodelianakis

Reporting Summary

Nature Research wishes to improve the reproducibility of the work that we publish. This form provides structure for consistency and transparency in reporting. For further information on Nature Research policies, see Authors & Referees and the Editorial Policy Checklist.

Statistical parameters

When statistical analyses are reported, confirm that the following items are present in the relevant location (e.g. figure legend, table legend, main text, or Methods section).

n/a	Confirmed	
☐	☒	The exact sample size (*n*) for each experimental group/condition, given as a discrete number and unit of measurement
☐	☒	An indication of whether measurements were taken from distinct samples or whether the same sample was measured repeatedly
☐	☒	The statistical test(s) used AND whether they are one- or two-sided<br>*Only common tests should be described solely by name; describe more complex techniques in the Methods section.*
☐	☒	A description of all covariates tested
☐	☒	A description of any assumptions or corrections, such as tests of normality and adjustment for multiple comparisons
☐	☒	A full description of the statistics including central tendency (e.g. means) or other basic estimates (e.g. regression coefficient) AND variation (e.g. standard deviation) or associated estimates of uncertainty (e.g. confidence intervals)
☐	☒	For null hypothesis testing, the test statistic (e.g. *F*, *t*, *r*) with confidence intervals, effect sizes, degrees of freedom and *P* value noted<br>*Give P values as exact values whenever suitable.*
☒	☐	For Bayesian analysis, information on the choice of priors and Markov chain Monte Carlo settings
☒	☐	For hierarchical and complex designs, identification of the appropriate level for tests and full reporting of outcomes
☒	☐	Estimates of effect sizes (e.g. Cohen's *d*, Pearson's *r*), indicating how they were calculated
☐	☒	Clearly defined error bars<br>*State explicitly what error bars represent (e.g. SD, SE, CI)*

Our web collection on statistics for biologists may be useful.

Software and code

Policy information about availability of computer code

Data collection	The flow cytometer raw data were collected using the BD Accuri C6 software and the optical density was measured at 600 nm using a Tecan Infinite M200 plate reader
Data analysis	For the analysis of the data we used R (boxplots and mixed effects models), PRIMER v 6 for Windows (Bray-Curtis similarity calculation and non-metric multidimensional scaling analysis) and Matlab R2018a (for Ordinary Differential Equation modeling).

For manuscripts utilizing custom algorithms or software that are central to the research but not yet described in published literature, software must be made available to editors/reviewers upon request. We strongly encourage code deposition in a community repository (e.g. GitHub). See the Nature Research guidelines for submitting code & software for further information.

Data

Policy information about availability of data

All manuscripts must include a data availability statement. This statement should provide the following information, where applicable:

- Accession codes, unique identifiers, or web links for publicly available datasets
- A list of figures that have associated raw data
- A description of any restrictions on data availability

Flow cytometry data are available in .fcs format online (http://flowrepository.org) under the “FR-FCM-ZYG6” and “FR-FCM-ZYTD” identifiers. The 16S rRNA gene

sequences of the representative isolates have been deposited in the GenBank database under accession numbers MH998420- MH998449. Modeling code is available at the personal webpage of AL (http://alexanderlorz.com/dispersal-dominates-over-selection.html).

Field-specific reporting

Please select the best fit for your research. If you are not sure, read the appropriate sections before making your selection.

☐ Life sciences ☐ Behavioural & social sciences ☒ Ecological, evolutionary & environmental sciences

For a reference copy of the document with all sections, see nature.com/authors/policies/ReportingSummary-flat.pdf

Ecological, evolutionary & environmental sciences study design

All studies must disclose on these points even when the disclosure is negative.

Study description	The aim of our study was to investigate the mechanism by which dispersal homogenizes a bacterial metacommunity. Our two hypotheses were that dispersal could homogenize a metacommunity indirectly and only when it weakens selection, or directly via the immigration of individuals without selection being weakened.<br>To test these two hypotheses, we designed a closed circulation system where medium was flowing among three incubation vessels, each one set at a different temperature. In this system dispersal is represented by the passive transfer of cells among the incubation vessels (so it can be manipulated by changing the speed of circulation) and selection by the temperature difference among the three vessels. When we increase the speed of circulation, dispersal increases and selection gradually decreases due to mixing. This system allowed us to increase dispersal while gradually weakening selection, thus enabling us to covariate dispersal and selection in a controlled way and test our two hypotheses.<br>We performed five experiments, each one at a different circulation speed, each one three independent times. We first grew the metacommunity at zero circulation speed, imposing only variable selection, and then at four different known speeds. Finally, we simulated the heat transfer and population dynamics of the system in 100 scenarios of intermediate dispersal using Ordinary Differential Equations to pinpoint exactly at which circulation speed, and how, dispersal effectively homogenizes the metacommunity.
Research sample	The subject of our study was a synthetic bacterial community composed of three strains; a Bacillus sp., a Staphylococcus sp. and a Chryseobacterium sp. strain, that we isolated from an arid soil in Thuwal, Saudi Arabia. We selected these three isolates because: 1) we could quantify their populations with great accuracy even when grown together, using staining-free flow cytometry with a temporal resolution of 30 minutes 2) their growth depended on temperature, so we could impose selection by growing them at different temperatures.
Sampling strategy	Our dependent variables were the populations of the three strains in the communities (in the experiments with no dispersal) and in the metacommunity (in the experiments with dispersal).<br>To safely estimate the mean values of our dependent variables, we first quantified the recording instument's (flow cytometer - BD Accuri C6) variability (coefficient of variation - CV) by repeatedly measuring the same six samples twelve times each. We then determined the required sampling depth (how much time each sample had to be read in the cytometer) so that the variability from three technical and three biological replicates is indistinguishable from the variability of the instrument (i.e., a CV of 3.75%). We found that in both cases, 90 seconds at "low" acquisition speed (i.e., 14 μl per minute) and a minimum of 50,000 recorded events was enough to reduce the variability in the estimation of our depended variables to the instrument's background levels.
Data collection	We recorded all experimental data in real time. We ran the samples in the flow cytometer within 20 minutes after sampling, and we kept the samples in ice before running them. To minimize the effect of a sample staying in ice on the depended variable (the population densities of the three strains) we analyzed one replicate from each sample category at a time, so that the effect is "split" when comparing the categories. We followed the same procedure when determining the instrument's variability. SF, AVC, AL and AB sampled and recorded the data jointly during the experiments.
Timing and spatial scale	Since ours is not a field study, this field is non-relevant. Nevertheless, we report that we performed all the main experiments between November and December of 2017 and between July and August, 2018.
Data exclusions	We excluded one biological replicate from the experiment without dispersal at 25 degrees Celcius because we erroneously inoculated the respective falcon tube with less starting inoculum. This exclusion had no apparent effect on the recorded within-replicate variability.
Reproducibility	We successfully reproduced the temperature dynamics of the closed circulation system in all different circulation speeds. We performed the experiments with sterile LB medium and sterile deionized water. We also successfully reproduced the growth assays without dispersal twice for the temperatures of 25, 37 and 42 degrees Celcius (nominal) and the experiments with dispersal twice at each circulation speed (three independent experiments at each circulation speed).
Randomization	The only randomizable parameter in our system was the position of the falcon tubes in the incubators (thermomixers). Even though this is not expected to affect our experiment because the heat transfer from the incubator is homogeneous, we placed the tubes at the different experiments in random positions each time.
Blinding	Blinding was not relevant in our study because it did not involve human participants or any parameters that were manually recorded and could be biased by the human researchers.

Did the study involve field work? ☐ Yes ☒ No

Reporting for specific materials, systems and methods

Materials & experimental systems

n/a	Involved in the study	
☒	☐	Unique biological materials
☒	☐	Antibodies
☒	☐	Eukaryotic cell lines
☒	☐	Palaeontology
☐	☒	Animals and other organisms
☒	☐	Human research participants

Methods

n/a	Involved in the study	
☒	☐	ChIP-seq
☐	☒	Flow cytometry
☒	☐	MRI-based neuroimaging

Animals and other organisms

Policy information about studies involving animals; ARRIVE guidelines recommended for reporting animal research

Laboratory animals	The subject of our study was a synthetic bacterial community composed of three strains; a Bacillus sp., a Staphylococcus sp. and a Chryseobacterium sp. strain.
Wild animals	The study did not involve wild anmals
Field-collected samples	The only field samples in our study were the soil samples where we originally isolated the bacterial strains from. These samples were collected aseptically from a garden plot and were transferred to the lab within half an hour, where the isolation commenced within one hour. The soil was kept at room temperature until that time.

Flow Cytometry

Plots

Confirm that:

☒ The axis labels state the marker and fluorochrome used (e.g. CD4-FITC).

☒ The axis scales are clearly visible. Include numbers along axes only for bottom left plot of group (a 'group' is an analysis of identical markers).

☒ All plots are contour plots with outliers or pseudocolor plots.

☒ A numerical value for number of cells or percentage (with statistics) is provided.

Methodology

Sample preparation	Since we performed staining-free flow cytometry, no sample preparation was required. We counted samples in LB medium sampled aseptically from our experiments in real time, or 1000-fold dilutions of overnight cultures in physiological solution. We ran in the cytometer samples from sterile LB medium and sterile physiological solution to deduct the recorded events from our calculations. The corresponding plots of the "noise" from the LB and the physiological solution are provided in the Supplementary Information.
Instrument	BD Accuri C6
Software	BD Accuri C6 software
Cell population abundance	We drew non-overlapping gates in the front/side scatter (FSC-A/SSC-A) plots that contained the majority of the events of samples from pure cultures. We defined these gates as “representative” and we used them to quantify the total populations of each strain in the synthetic community, correcting for "spillover" of one population to the other two “representative” gates.
Gating strategy	Please see above, we only gated our populations on the FSC-A/SSC-A axes. regarding the box below, the "representative" gating is described both in Materials & Methods and in the Supplementary Information

☒ Tick this box to confirm that a figure exemplifying the gating strategy is provided in the Supplementary Information.
